# Bio-Synthesis of *Aspergillus terreus* Mediated Gold Nanoparticle: Antimicrobial, Antioxidant, Antifungal and In Vitro Cytotoxicity Studies

**DOI:** 10.3390/ma15113877

**Published:** 2022-05-29

**Authors:** Rahul Chandra Mishra, Rishu Kalra, Rahul Dilawari, Mayurika Goel, Colin J. Barrow

**Affiliations:** 1 TERI-Deakin Nano Biotechnology Centre, The Energy and Resources Institute (TERI), TERI GRAM, Gurgaon 122001, India or rcmishra@deakin.edu.au (R.C.M.); kalrar@deakin.edu.au (R.K.); mayurika.goel@teri.res.in (M.G.); 2 Centre for Bioprocessing, School of Life and Environmental Sciences, Deakin University, Waurn Ponds, VIC 3220, Australia; 3 CSIR-Institute of Microbial Technology, Sector-39-A, Chandigarh 160036, India; rahuldilawari1@gmail.com

**Keywords:** endophytic fungi, *Aspergillus* sp., gold nanoparticles, antibacterial, antifungal

## Abstract

Gold nanoparticles (GNP) were bio-fabricated utilizing the methanolic extract of the endophytic isolate *Aspergillus terreus*. The biosynthesised gold nanoparticles (GNP023) were characterised using UV-visible spectroscopy (UV-Vis); transmission electron microscopy (TEM), Fourier-transform nfrared spectroscopy (FTIR) and X-ray diffraction (XRD) studies. The bio-fabricated GNP023 displayed a sharp SPR peak at 536 nm, were spherically shaped, and had an average size between 10–16 nm. The EDX profile confirmed the presence of gold (Au), and XRD analysis confirmed the crystalline nature of GNP023. The antimicrobial activity of GNP023 was investigated against several food-borne and phytopathogens, using in vitro antibacterial and antifungal assays. The maximum zone of inhibition was observed for *S. aureus* and *V. cholera* at 400 μg /mL, whereas inhibition in radial mycelial growth was observed against *Fusarium oxysporum* and *Rhizoctonia solani* at 52.5% and 65.46%, respectively, when challenged with GNP023 (200 μg/mL). Moreover, the gold nanoparticles displayed significant antioxidant activity against the ABTS radical, with an IC_50_ of 38.61 µg/mL, and were non-toxic when tested against human kidney embryonic 293 (HEK293) cells. Thus, the current work supports the application of myco-synthesised gold nanoparticles as a versatile antimicrobial candidate against food-borne pathogens.

## 1. Introduction

Metallic nanoparticles synthesised through a biological route constitute an active area of application research. Amongst the various nanoparticles of metallic origin, gold nanoparticles have emerged as potential candidate in biomedical research, optical research, cosmetics and other areas. Biologically sythnthesised gold nanoparticles are well-known for displaying diverse biological activities such as antibacterial, anticoagulants, antidiabetics, antrioxidanes, antifungal, catalytic and thrombolytic activity [1,2], and are, also, non-toxic with high absorbance. According to a report by the WHO, diseases due to foodborne pathogenic bacteria are a major cause of infection every year, and it is estimated that 25% of harvested agricultural products are affected by phytopathogens [3]. An important area is the losses faced, due to phytopathogens and food-borne pathogens during postharvest storage and handling/distribution. Food-borne pathogenesis is a well-established health hazard, especially for infants. Some of the most important communicable food-borne pathogens are *Salmonella* sp. [4]; *Listeria* sp. [5]; *Escherichia* *coli* O157 [6]; *Vibrio cholera* El Tor strain N16961 [7]; and *Clostridium* sp. [8]. Endophytic fungal bioactive compounds have been reported to have proven biocontrol abilities, in the food and agriculture sectors [9,10,11].

The antimicrobial potential of gold nanoparticles against food-borne human and plant pathogens has been well documented [12,13]. Chemically synthesised metallic nanoparticles have been employed for the efficient management of phytopathogens [14,15]. However, reports have shown the environmental toxicity of chemically synthesised gold nanoparticles [16,17]. Instead, mycogenic metallic nanoparticles are relatively safe, compared to their synthetic counterparts, and display several modes of inhibitory mechanisms against both plant and food-borne pathogens [18,19]. Gold nanoparticle synthesis, using endophytic fungal substrate, is advantageous compared to other microbial counterparts, as the fungal mycelium facilitates a mesh-like scaffold, which can withstand agitation and pressures in bioreactors and/or closed chambers. Some recent works have demonstrated the utilization of fungal isolate *Verticillium* sp. for the synthesis of gold nanoparticles [20]. There are limited reports of non-toxic myco-synthesised gold nanoparticles being effective against food-borne pathogens and phytopathogenic fungi [21].

The present work investigated the bio-fabrication of monocrystalline gold nanoparticles, using extracts of the endophytic fungus *Aspergillus terreus* AREF023, obtained from the surface-sterilised roots of *Datura metel,* which is known to possess a range of bioactivities. To the best of our knowledge, this is the first report of the bio-fabrication of gold nanoparticles utilizing endophytic fungi *A. terreus,* with these nanoparticles exhibiting antibacterial, antifungal, antioxidant and cytotoxicity activity.

## 2. Materials and Methods

### 2.1. Chemicals and Materials Used

Chloroauric acid (HAuCl4), Ethidium Bromide (EtBr), MTT (3-[4,5-dimethylthiazol-2-yl]-2,5 diphenyl tetrazolium bromide) (for in-vitro cytotoxicity assay), Vancomycin and Chloramphenicol were procured from Sigma Aldrich (St. Louism, MO, USA). The reagents used were of the highest analytical grade available.

### 2.2. Preparation of Crude Extract of A. terreus AREF023

Endophytic fungal isolate *A. terreus* AREF023, obtained from the roots of *Datura metel* (biodiversity park situated in the Aravalli region (28°28′59.9″ N 77°06′34.7″ E), Gurugram, Haryana, India) was previously isolated in our laboratory [22]. The fungal isolate was cultured and extracted, as previously reported [23]. The isolate AREF023 was inoculated in a 2000 mL Erlenmeyer flask, with 500 mL of potato dextrose liquid medium. The flasks were incubated for 10 days, at 26 °C and 180 rpm, in the dark. The culture filtrate and mycelia were separated, with the help of Whatman filter paper No. 1 extensively washed using distilled water to eliminate the media traces, and extracted with 70% methanol *v/v* twice. The methanolic crude was, then, concentrated using a rotary evaporator, diluted and lyophilised. The crude powder, thus obtained, was further used for the study.

### 2.3. Biosynthesis of Gold Nanoparticle GNP023

The aqueous solution of 1 mM aqueous hydrogen tetrachloroaurate (III) (HAuCl_4_.3 H_2_O, Sigma Aldrich, St. Louism, MO, USA) was prepared in double ionised water in a sterile amber-coloured bottle at room temperature. The different parameters for the synthesis of gold nanoparticles, such as pH, temperature and concentration of fungal extract, were optimised. The effect of pH on the synthesis of gold nanoparticles was evaluated, by preparing HAuCl_4_ solution at pH 4, 6, 8, 10 and 12. The effect of temperature on the reaction was studied at 25 °C, 40 °C, 50 °C and 60 °C, where the reaction temperature was maintained using a water bath. The concentration of fungal extract, in the range of 10 ppm, 25 ppm, 50 ppm, 100 ppm and 200 ppm, was used to find out the optimum concentration of the fungal extract for reducing HAuCl_4_. For the optimal reduction of gold salt, fungal crude extract (100 ppm) was mixed with 1 mM aqueous HAuCl_4_ solution in a 500 mL conical flask, at 40 °C for 24 h, in an orbital shaker at 150 rpm. The pH of the reaction mixture was maintained at 8.0.

### 2.4. Characterization Studies of the Synthesised Gold Nanoparticle GNP023

#### 2.4.1. UV-Visible Spectroscopy and Light Scattering (DLS) Study

A Shimadzu UV-1800 (Kyoto, Japan) UV-visible spectrophotometer was employed, to detect the surface plasmon resonance (SPR) signatures and different parameters, for the optimised bio-fabrication of gold nanoparticles between 350 to 800 nm. A piece of dynamic light-scattering equipment Zetasizer Nano-ZS, Malvern Instruments Ltd. (Malvern, UK) was used to analyse the size and zeta potential of the gold nanoparticles GNP023.

#### 2.4.2. Fourier-Transform Infrared Spectroscopy (FTIR)

A Nicolet 6700 (Thermo Fischer Scientific, Waltham, MA, USA) FTIR instrument was employed to characterise the functional groups and surface capping of the purified air-dried gold nanoparticle GNP023, using 64 average scans in the range of 400–4000 cm^−1^.

#### 2.4.3. Transmission Electron Microscopy (TEM)

The morphological identification details of the gold nanoparticles GNP023 was analysed using a transmission electron microscope (TECNAI G2 T20 TWIN, FEI Company, Eindoven, The Netherlands). The elemental composition of the GNP023 was analysed using the Energy-dispersive X-ray detection instrument (EDX) (EDAX Inc., Tilburg, The Netherlands). Five milligrams of pure dried GNP023 dissolved in 500 µL deionised water was briefly sonicated for the dissolution of the nanoparticles. About ~10 mL of the sonicated sample was drop casted and air dried on a carbon-coated copper grid, in the dark. The EDX spectrum and TEM images were recorded and analysed, employing the processed grid at 200 kV accelerated voltage.

#### 2.4.4. X-ray Diffraction (XRD) Analysis

The crystalline nature of the synthesised gold nanoparticle GNP023 was determined using X-ray diffraction (XRD) analysis. The XRD pattern of the pure powdered GNP023 was assessed on a MiniFlex-600 benchtop XRD instrument (Rigaku, Kyoto, Japan). The instrument was operated at 40 kV volts, 15 mA current and with a Cu kα radiation in a θ–2θ configuration. The diffracted intensities were recorded from 10 to 90 degrees at 2 degree angles, per Scherrer’s formula:

D = 0.94 λ/β Cos θ

was used to determine the crystallite size of the biosynthesised GNP023, based on the width of the XRD peaks. Where, D-Average crystallite domain size was perpendicular to the reflecting planes; λ-Wavelength; β-Full width was at half maximum (FWHM) and a θ-diffraction angle.

#### 2.4.5. UPLC–MS Analysis of the *A. terreus* AREF023 Biomass

A high-definition mass spectrometry SYNAPT G2 instrument and an ultra-performance ACQUITY LC (WATERS, Bangalore, India), 2.1 × 50 mm column, ACQUITY UPLC, C 18 II column (WATERS, Bangalore, India) was employed to perform the high-resolution LC-ESI-MS, as previously demonstrated [24]. Briefly, for the dried methanolic extract of *A. terreus* AREF023 isolate; water (0.1% formic acid)—acetonitrile gradient system: 2% acetonitrile, linearly increased to 90% in 30 min, with a holding time of 5 min, starting from 2% acetonitrile for 2 min and increasing to 100% in 30 min, keeping it for 5 min, was employed for the MS system at a positive ESI mode. The instrument was adjusted to a resolution > 6000.

### 2.5. Bioactivities of Gold Nanoparticle GNP023

#### 2.5.1. Antibacterial Activity of Gold Nanoparticles GNP023

The antibacterial efficacy of the biosynthesised GNP023 was assessed against the bacterial pathogens. *Salmonella typhimurium* (MTCC 734), *Staphylococcus aureus* (MTCC737), methicillin-resistant *staphylococcus aureus* (MRSA) (ATCC BAA811) and *Vibrio cholerae* (N16961) were procured from the Microbial Type Culture Collection (MTCC), IMTECH, Chandigarh, India, using the single-disc diffusion method. The test pathogens were seeded in nutrient broth (NB), to reach about 3.0 × 10^−8^ CFU/mL overnight. The overnight seeded cultures were, then, transferred to fresh NB medium and grown till 1.5 × 10^−8^ CFU/mL (0.5 McFarland scale), and 100 μL of this culture was spread over Muller Hinton Agar (MHA) plates. Sterilised 6 mm Whatman paper No. 1 discs were, aseptically, placed on MHA plates with test cultures. Five microliters (5 µL) of GNP023, prepared in 10% DMSO at different concentrations (400, 200 and 100 µg/disc), as well as AREF023 methanol extract (ME) at concentrations (800, 600 and 400 µg/disc), were loaded, aseptically. Standard antibiotics Vancomycin (S. aureus and MRSA), Chloarmphenicol (*S. typhimurium* and *V. cholera*) and 10% DMSO served as controls. The plates were incubated at 37 °C for 24 h and observed for a clear zone of inhibition (in mm). The antibacterial assay was performed in triplicate, and the quantitative variables were represented in terms of mean ± SD [25].

#### 2.5.2. Antifungal Activity of Gold Nanoparticles GNP023 against Phytopathogens

Two plant-pathogenic fungi, *Fusarium oxysporum* (ITCC 6709) and *Rhizoctonia solani* (AG1-IA), were obtained from the Indian Type Culture Collection (ITCC), Division of Plant Pathology, ICAR-Indian Agricultural Research Institute, New Delhi. Antifungal activity of the GNP023 was evaluated, in accordance with the previously described method of Aguilar-Mendez et al. [26]. Briefly, the mentioned phytopathogens were propagated in Potato Dextrose Agar (PDA) medium and were incubated for 7 days at 27 ± 1 °C. Different concentrations of GNP023 (25, 50, 100, 200 μg/mL of PDA medium) in colloidal solution were prepared and added to the sterilised PDA medium, along with a suitable control (PDA medium only). Pathogenic fungal mycelial plugs of 3 mm diameter were punched out and placed on the center of the PDA plates amended with the gold nanoparticles. The plates were incubated at 27 ± 1 °C. The experiment concluded when the mycelium of fungi reached the edges of the control plate. The resulting fungal colonies were measured and the antifungal index (AI) was determined. The antifungal assay was performed in triplicate and the quantitative variables were represented in terms of ±SD.

#### 2.5.3. ABTS Scavenging Activity

The 2,2′-azino-bis(3-ethylbenzothiazoline-6-sulfonic acid (ABTS) scavenging activity of the gold nanoparticle GNP023 was performed, in accordance with the method described by Re and collaborators [27]. ABTS solution was prepared by reacting 7 mM ABTS salt with 2.45 mM potassium persulfate solution in equal volumes. The reaction mixture was incubated for 12 h at room temperature, in the dark, and diluted to an absorbance of 0.70 ± 0.02 at 745 nm. The sample, at varying concentrations (2.5–50 µg/mL), was mixed with a test solution to a final volume of 1 mL, and the absorbance was monitored at 734 nm after 6 min. Commercial standards Trolox, Ascorbic acid and tert-Butylhydroquinone (TBHQ) were, also, compared. The scavenging capability was calculated using the following equation:

Scavenging Effect (%) = (A_0_ − A_s_)/A_0_ × 100

(A_0_—absorbance of the blank reaction; A_s_—absorbance of the sample).

#### 2.5.4. DNA Nick Assay

Antioxidant activity of the gold nanoparticles GNP023 was, also, determined with the DNA nicking assay, performed in accordance with our previously described method [24], with slight modifications. Briefly, pBSK DNA and GNP023, in varying concentration (25–200 μg/mL), as well as AREF023 methanol extract at concentrations (100–300 μg/mL), were incubated for 10 min at 25 °C, with the subsequent addition of an equal volume of Fenton’s reagent. The reaction mixture was, then, incubated for 30 min at 37 °C. Curcumin was used as a positive control. The electrophoretic mobility of the test DNA was examined using 1% agarose ethidium bromide gel.

#### 2.5.5. Cell Cytotoxicity/Viability Assay

The cytotoxicity of the gold nanoparticle GNP023 was assessed towards the HEK 293 T cell line using MTT (3-[4,5-dimethylthiazol-2-yl]-2,5 diphenyl tetrazolium bromide) assay [28]. Log phase cells were harvested and adjusted to (1 × 104/well) cell counts in DMEM medium, supplemented with 10% fetal bovine serum (FBS), in 5% CO_2_ at 37 °C, in a 96-well plate for 12 h. Next, the cells were treated with different concentrations of gold nanoparticles GNP023, as was the AREF023 methanol extract, and incubated for 24 h at 37 °C. Then, a filtered MTT reagent (50 mg/mL) solubilised in culture medium was added to the cells and further incubated for 6–8 h, under 5% CO_2_ at 37 °C. The formazan crystals were dissolved using 100 µL of DMSO (molecular grade). Absorbance was measured at wavelength 570 nm, using a Power Wave HT Microplate Spectrophotometer (BioTek, Winooski, VT, USA) that was used for spectrophotometric measurements. The percent difference between treated and untreated cells (% Viability) was calculated by the following formula:

% Viability = (absorbance of treated sample/absorbance of untreated) × 1

## 3. Results and Discussion

### 3.1. Bio-Fabrication of Gold Nanoparticles from A. terreus AREF023

Endophytic fungi are a ubiquitous source of natural bioactive compounds, having potential application in the pharmaceutical, food and health sectors. Moreover, they are widely used in the synthesis of nanomaterials and nanoparticles [28]. In this study, we have shown the potential of the endophytic isolate *A. terreus* AREF023 (obtained from roots of *D. metel* plant), in the bio-fabrication of gold nanoparticles. The identity of the fungus was established by 18 S rDNA and ITS analysis (Gen Bank accession no.: MT013407). The methanolic extract of *A. terreus* AREF023 was treated with chloroauric salt, and the bio-reduction of the Au+ ions to Au nanoparticles was observed.

### 3.2. Characterization Studies of the Synthesised Gold Nanoparticle GNP023

#### 3.2.1. UV-Visible Spectroscopy

**Figure 1:**
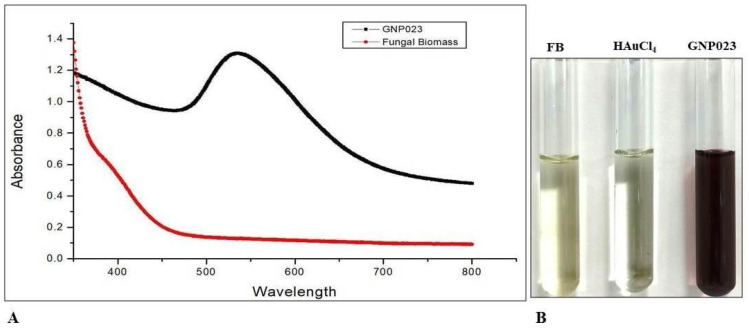
(**A**) UV-absorption *spectra* of gold nanoparticles GNP023 for reaction of the fungal extract with *HAuCl_4_ solution* and (**B**) comparison between nanoparticles synthesis by HAuCl_4_ solution and the biomass of *A. terreus* AREF023.

The change in colour of the fungal-extract-challenged metal solution was used as a preliminary indicator of gold nanoparticle synthesis. Gold nanoparticle formation was confirmed by the visual colour change, light yellow to pink ruby red (Figure 1B), which indicated variation in the oxidation states of metal ions. In this reaction, Au+ was reduced to Au° by the bioactive molecules present in the extract of the endophytic isolate *A. terreus* AREF023. The change in colour was due to the excitation of surface plasmon resonance of the gold nanoparticles [29]. The UV-Vis SPR peaked at 536 nm, which increased as a function of time without effecting the peak position, indicating the typical bio-formation of gold nanoparticles. This observation further validates the conversion of gold ions into gold nanoparticles. A similar observation was demonstrated by Raouf et. al., where reddish AuNPs were formed using the ethanolic extract of *G. elongata* [30]. The synthesised nanoparticles showed a dark-pink-ruby-red colour and a high-peak plasmon band between 510–560 nm.

The findings are in good agreement with previously published work [31]. This result suggests that *A. terreus* AREF023 mediated the biosynthesis of mono-dispersed gold nanoparticles. SPR peaks of the metallic nanoparticle are dependent on the size, shape and reaction conditions. In this study, the SPR peak retained its spectral position, confirming the uniform distribution of the size of the nanoparticles (Figure 1). There have been numerous reports of silver and gold nanoparticles being synthesised using *Aspergillus* sp., such as *Aspergillus oryzae* [32], *Aspergillus niger* [33], *Aspergillus fumigatus* [34] and *Aspergillus flavus* [35], demonstrating potential activity against human pathogens and phytopathogens.

**Figure 2:**
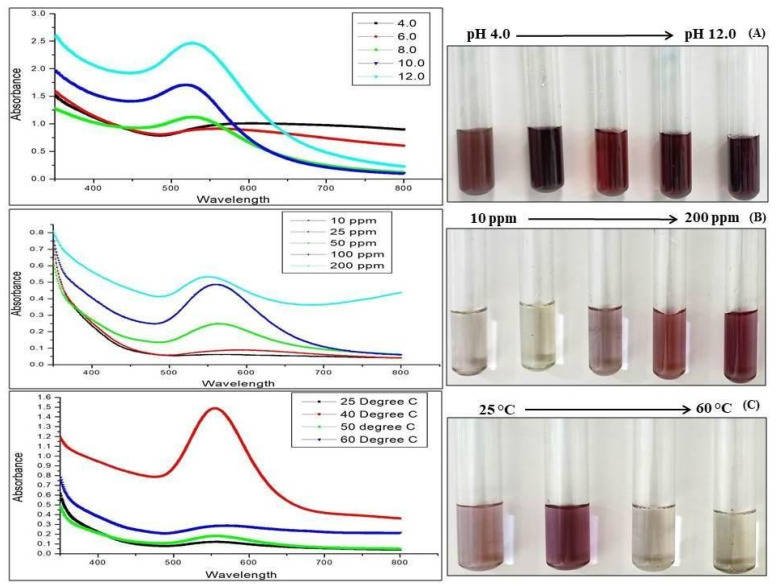
(**A**) Effect of pH on GNPs production, (**B**) effect of fungal biomass concentration on GNPs production and (**C**) effect of temperature on GNPs production.

With increase in pH, the colour of the reaction changed. As shown in the UV-visible spectra (Figure 2A), the absorbance increased as a result of the increase in surface plasmon resonance. The change in colour appeared as deep pink, and a sharp peak was observed from pH 8.0 onwards, indicating the presence of gold nanoparticles, while broad peaks were observed at lower pH values. pH, also, affects the overall charge of the particle, thus affecting its capping property and stability. Therefore, pH 8.0 was shown to be the most favorable pH for GNPs biosynthesis. Figure 2B shows the formation of GNP023, by increasing the fungal biomass concentration from 10–200 ppm. The UV-visible spectra reflected the shift in peaks and visible change in the colour of the reaction beyond 100 ppm concentration, whereas broad peaks were observed at lower concentrations. Therefore, 100 ppm was the optimum concentration for gold nanoparticle biosynthesis. Figure 2C shows the biosynthesised GNPs absorption spectra, at various temperatures ranging from 25 °C to 60 °C. The rate of gold-salt reduction was enhanced by increasing the temperature of the reaction, as illustrated by the quick change in the solution colour, and increasing the absorbance intensity gave the maximum peak at 550 nm. Due to the rapid reaction rate at an elevated temperature, an increase in the kinetic energy of the molecules occurred, and the Au ions received energy more rapidly, resulting in decreased particle-size growth. Hence, the elevated temperature resulted in the formation of smaller and uniform size distributed particles. The above data confirm the formation of gold nanoparticles at 40 °C, whereas at an elevated temperature no visible change in colour or sharp peak was observed.

#### 3.2.2. TEM and EDAX Analysis of the Gold Nanoparticle GNP023

**Figure 3:**
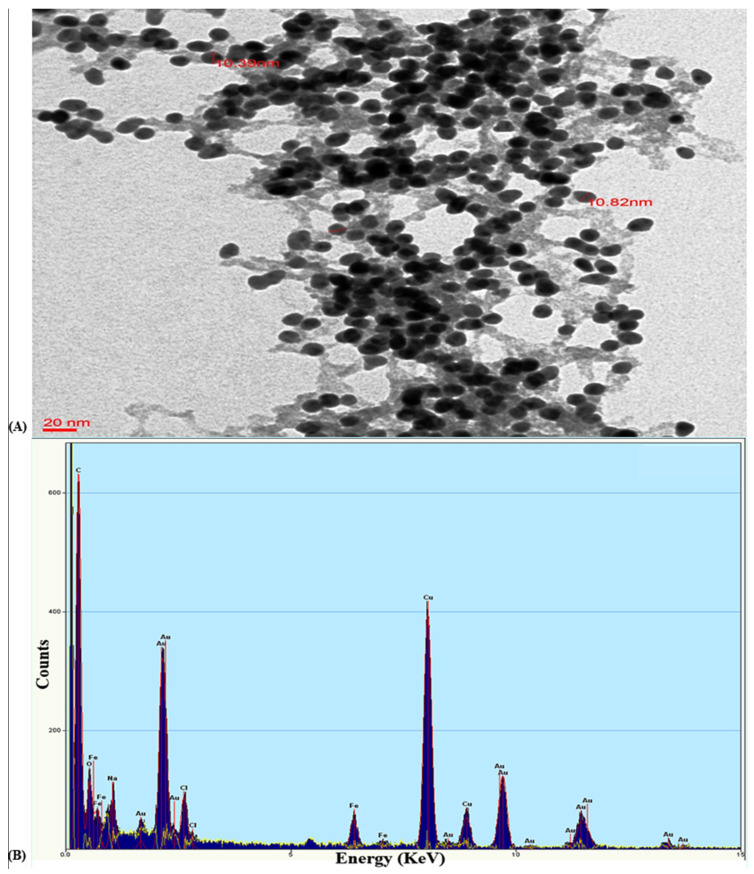
(**A**) Transmission electron micrograph of gold nanoparticle GNP023 and (**B**) EDX spectrum, confirming a sharp peak in the range of 1–5 keV, confirming the presence of gold. In both (**A**) and (**B**) EDX spectrum the largest peak is C (at about 0.5 KeV), second largest is Cu (at about 8 KeV), and third largest is Au (at about 2 KeV).

The transmission electron microscopic image (Figure 3A) recorded different sizes of the gold nanoparticles, which were synthesised using the optimised conditions. Our findings showed that spherical structures of the gold nanoparticles were formed in the reaction solution. The average sizes of these gold nanoparticles were measured, and the size was in the range of 9–14 nm. The average size of GNP023 was 10 ± 1 nm. Similar results were observed in a study, where the authors demonstrated spherical gold nanoparticles with average size ~15 nm diameters, from the bio-extract of *P. valderianum* [36]. The gold nanoparticles formed in this study were small and spherical, whereas previously reported particles were, primarily, irregular or triangular and tended to be of larger size (e.g., greater than 20 nm) [30,32,37,38].

The elemental composition employing EDX analysis (Figure 3B) shows the presence of a strong signal, corresponding to the gold atoms. Furthermore, the sharp absorption peak between 1–5 keV is typical for the absorption of gold nanoparticles [39]. This observation, clearly, indicates that these nanoparticles were, exclusively, composed of elemental gold. Nevertheless, the presence of mixed precipitates from the fungal filtrate was indicated by the presence of EDX peaks, corresponding to C, O, K, Cl and N.

#### 3.2.3. DLS Study of the Gold Nanoparticle GNP023

**Figure 4:**
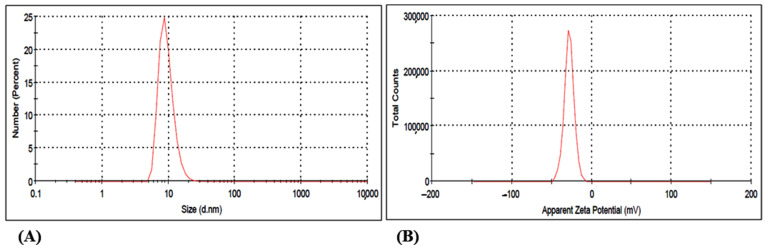
Dynamic light scattering study representing (**A**) size distribution (Average size −45.53 nm) and (**B**) zeta potential (−28.2).

The zeta potential and average size of the biosynthesised gold nanoparticles GNP023 were found to be −28.2 mV and 45.53 ± 0.12 nm, respectively (Figure 4A,B). A low polydispersity index (PDI) value, of 0.271, indicates high particle homogeneity. Whereas the size range below 100 nm points out the dimensional correctness, the high zeta potential assured the stability of the gold nanoparticles GNP023. Sujitha et al. demonstrated similar results, with an average size of gold nanoparticles in the range of 30–60 nm, from the aqueous extracts of citrus fruits *C. limon, C. reticulata* and *C. sinensis* [40].

#### 3.2.4. FTIR Analysis of the Gold Nanoparticle GNP023

**Figure 5:**
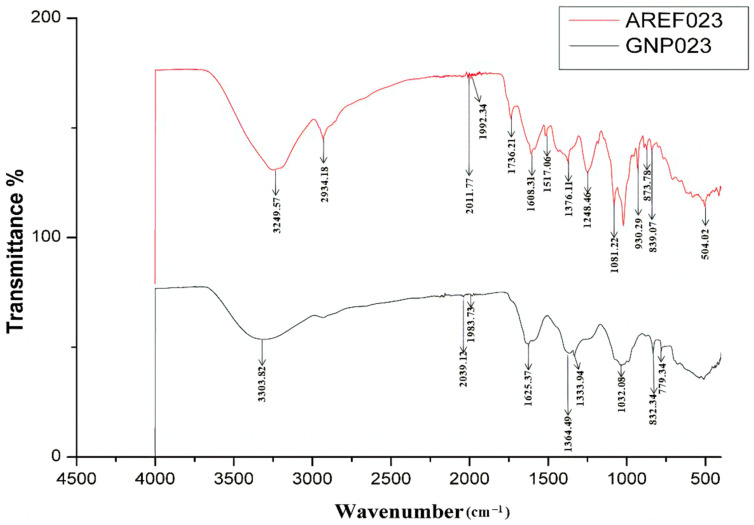
FTIR analysis spectra of *A. terreus* AREF023 extract and gold nanoparticles GNP023.

The presence of functional groups in the fungal extracts, which accounts for the bio-reduction and stabilization of the gold nanoparticles, were studied with the help of the FTIR spectrum, obtained in the range of 400 cm^−1^ to 4000 cm^−1^ (Figure 5). The peaks obtained for the AREF023 methanolic extract were at 3249 cm^−1^ and 2934 cm^−1^, whereas GNP023 exhibited peaks at 3303 cm^−1^. The strong broad peak at 3303 cm^−1^ signifies the presence of OH bonded stretch, signifying the presence of a carboxylic group. The peak at 2039 cm^−1^ and 1983 cm^−1^ signifies the presence of C–H bending stretch, in plane deformation with aromatic ring stretching. Other significant bands appearing after bio-reduction with HAuCl4 were at 1625 cm^−1^, 1364 cm^−1^ and 1333.94 cm^−1^, corresponding to C=O stretching vibrations and phenolic OH bending, respectively. This confirms the presence of ketone and alcohol-functional-group capping of the gold nanoparticle. Further, peaks at 1032 cm^−1^ correspond to C–N stretching, confirming the presence of amines, whereas the peaks at 832 cm^−1^ and 779 cm^−1^ correspond to C–H bending planar vibrations.

Our findings are in agreement with Singh et al. [41], who confirmed that proteins bind to metallic nanoparticles through amines as well as electrostatic interactions. The comparative shifts in the FTIR spectra might be due to the interaction of the chemical moieties of the fungal extract with the metallic scaffold. Moreover, the carboxylic acids, phenolic acids and amides in the enzymatic proteins of the fungal methanolic extract are, probably, important for both the bio-reduction and stabilization of GNP023. The results obtained here are in good agreement with previous reports of *Aspergillus niger* and *Trichoderma longibrachiatum* enzyme mediated-gold-nanoparticle synthesis, describing the involvement of fungal proteins in the bio-stabilization and capping of the gold nanoparticles [1].

#### 3.2.5. XRD Characterization of the Gold Nanoparticle GNP023

**Figure 6:**
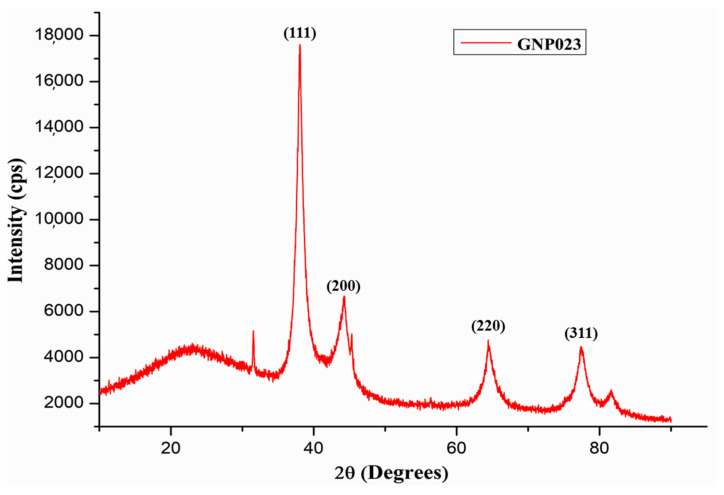
XRD patterns of bio-synthesized gold nanoparticles GNP023.

The crystalline nature of the biosynthesised gold nanoparticle GNP023 was confirmed by X-ray diffraction analysis. The XRD pattern obtained from the gold nanoparticles is shown in Figure 6. The Bragg’s reflections obtained from the synthesised nanoparticles, clearly, correspond to the face-centred cubic (fcc) crystalline structure of gold. The four distinctive diffraction peaks, corresponding to the (111), (200), (220) and (311) planes of fcc gold, emerging at 2θ = 38.030, 44.30, 64.410 and 77.400 of metal gold, respectively (JCPDS 04-0784), indicate the presence of pure crystalline gold [37]. As per our findings, a very intense Bragg’s reflection was observed at (111), suggesting that the nanoparticles were lying flat on the planar surface, whereas the reflections corresponding to (220) and (311), with lattice spacing of 1.44 and 1.23 A°, were specific for spherical morphology, respectively. Further, the average crystallite size of the biosynthesised GNP023 was calculated, using the Debye–Scherer’s equation, as 16.00 nm. These results confirm the predominantly formed GNPs with (111) facets are consistent with the reported electron diffractions. Similar results were reported in a recent study of clove extracts [37].

### 3.3. UHPLC–MS Profiling of the Compounds Present in the Extract of A. terreus AREF023

**Figure 7:**
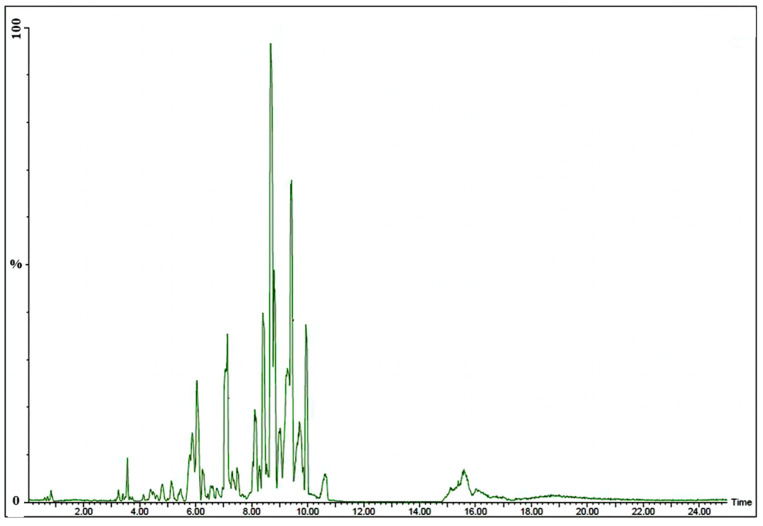
UHPLC Chromatograms of extract of *A. terreus* AREF023.

To study the chemical composition of the methanolic extract of *A. terreus*, AREF023 was subjected to LC-ESI-MS. Figure 7 displays the total ion current chromatogram (TIC) of AREF023 extract. Based on the *m*/*z* ratio of molecular ion [M + H]+, metabolites were tentatively identified, with MS and MS/MS spectral data shown in Table 1. The chromatogram of the AREF023 extract showed a peak at Rt 9.71 with *m*/*z* 799.6769 and chemical formula C_15_H_11_I_4_NO_4_, which was identified as Levothyroxine sodium anhydrous. A steroidal saponin compound, which is a well-known plant secondary metabolite and anticancer pharmaceutical bioactive obtained from endophytic fungi, was tentatively identified [42,43]. The peak at retention time 9.26, having *m*/*z* 815.6778 matching the chemical formula C_51_H_90_O_7_, was identified as Sitoindoside I. Other bioactive molecules were, also, present in the methanolic extract. The peak at Rt 8.1, with *m*/*z* 515.3141 with molecular formula C_34_H_42_O_4_, was determined as Flavidulol C, a well-known gernayl phenolic compound and antifungal against plant pathogens [44,45]. Similarly, the peak corresponding to Rt 7.04, with *m*/*z* 871.4661 and molecular formula C_44_H_70_O_17_, was identified as Capsicoside C3, a furostanol saponnin and well-known food additive, antimicrobial and antioxidant compound, having a myriad of pharmaceutical applications [46,47]. A carbamic acid ester, tert-Butyl {5-[6-(benzyloxy) naphthalen-2-yl]-2,2-dimethyl-1,3-dioxan-5-yl} carbamate, was tentatively identified at Rt 6.04 with *m*/*z* 464.2419 and molecular formula C_28_H_33_NO_5_. A peak at retention time 3.56 and *m*/*z* 702.6394, having molecular formula C_45_H_83_NO_4_, was identified as a benzamide molecule, N-(2-Hydroxyethyl)-3,5-bis(octadecyloxy)benzamide. These benzamide moieties obtained from endophytes are well known for their antimicrobial and anticancer properties [48]. The presence of these bioactive molecules in the methanolic extract further bolsters the pharmaceutical potential and applicability of this extract as a nano-formulation.

### 3.4. Bioactivities of the Synthesised Gold Nanoparticle GNP023

#### 3.4.1. Antimicrobial Activity of the Gold Nanoparticle GNP023

**Figure 8:**
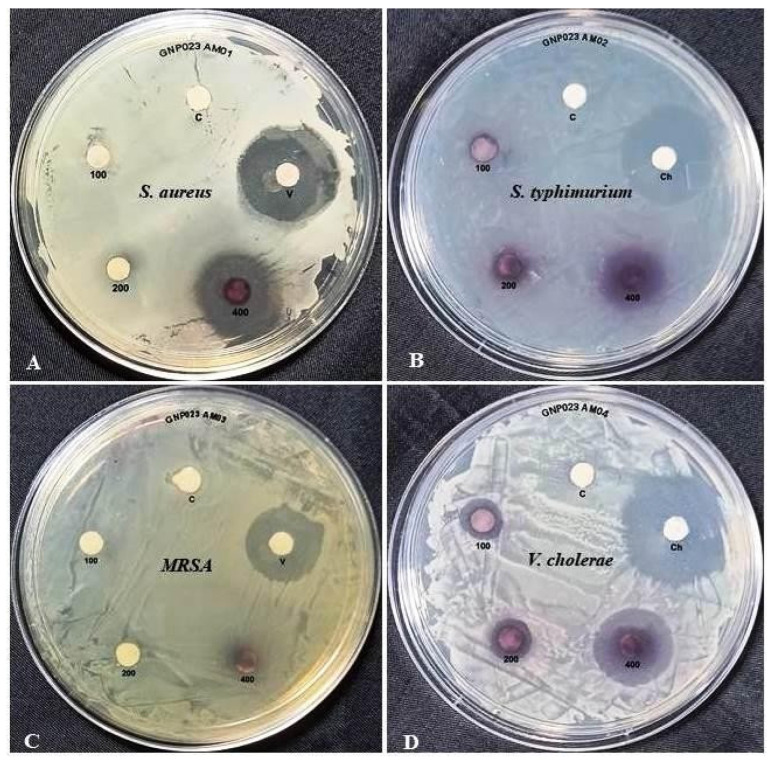
Zones of inhibition produced by GNP023 against bacterial pathogens (**A**)—*S. aureus*, (**B**)—*S. typhimurium*, (**C**)—Methicillin resistant *S. aureus* (MRSA) and (**D**)—*V. cholerae.* [V–Vancomycin; Ch—Chloramphenicol; 100—GNP023 100 µg/mL; 200—GNP023 200 µg/mL; 400—GNP023 400 µg/mL; C—Control].

Antibacterial activity of the biosynthesised gold nanoparticle GNP023 was assessed using the single-disc diffusion method [49]; notable zones of inhibition (ZOI) were observed against the bacterial pathogens *S. aureus* and *V. cholera.* No visible zone of inhibitions was observed against Methicillin resistant *S. aureus* (MRSA) or *S. typhimurium* (Figure 8). Maximum ZOI was observed in the case of *S. aureus* and *V. cholera* at 400 μg /mL, in comparison to the positive control (Table 2) (Figure 8). In contrast, the AREF023 methanol extract did not show any significant inhibition against these bacterial pathogens (provided as supplementary materials—Figure S1 and Table S1). Nobel metallic nanoparticles kill bacteria by divergent mechanisms, such as membrane disruptions, reactive oxygen species (ROS) generation and DNA damage as well as the degradation of some vital enzymes [50,51]. Among the different mechanisms discussed, ROS can be considered as the predominant mechanism that kills the bacteria, by disrupting the cell membranes, nucleic acids and proteins [52,53]. The current findings are in congruence with the previously reported results [54].

#### 3.4.2. Evaluation of Antifungal Activity of Gold Nanoparticles GNP023

**Figure 9:**
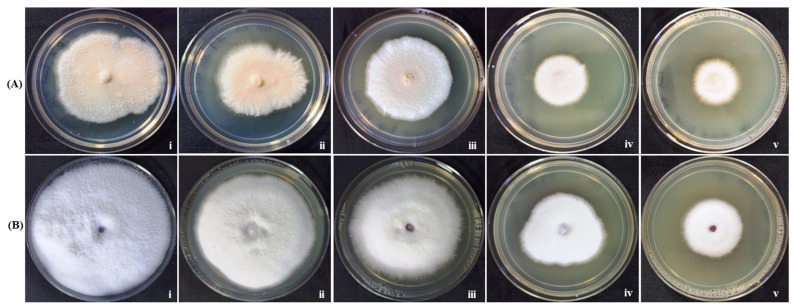
Antifungal activity of GNP023 s against plant pathogenic fungi, (**A**) *Rhizoctonia solani* and (**B**) *Fusarium oxysporum*. PDA plates were amended with different concentrations of GNP023 s: (i) control, (ii) 25 μg/mL, (iii) 50 μg/mL, (iv) 100 μg/mL and (v) 200 μg/mL of gold nanoparticles GNP023.

The radial mycelial growth of the two phytopathogenic fungal strains, *Fusarium oxysporum* and *Rhizoctonia solani*, was, differentially, inhibited by the tested gold nanoparticles GNP023 at different concentration levels (25 μg/mL, 50 μg/mL, 100 μg/mL, 200 μg/mL). The gradual effect of GNP023 concentrations that challenged the mycelial growth of the phytopathogens tested, after seven days of incubation, can be observed in Figure 9 and Table 3. Maximum inhibition of 52.5% and 65.46% was observed against *Rhizoctonia solani* and *Fusarium oxysporum* at the concentration of 200 μg/mL, respectively, whereas no significant inhibition was observed for the AREF023 methanol extract (provided as Supplementary Materials—Figure S2 and Table S2). These results were in agreement with the previously reported studies [2]. The gold nanoparticles display size-dependent fungicidal activity against the plasma-membrane proteins [55]. These gold nanoparticles interact, directly, with the fungal enzymes controlling the proton gradient across the plasma membrane. The fungal membranes, then, become incapable of channeling H+-ATPase transport, which, in turn, ceased cell growth [56]. In another report, gold nanoparticles were reported to diffuse through the fungal membranes and interact with the sulphur-conjugated proteins and/or the phosphate bases in the nucleic acids, resulting in the inhibition of DNA synthesis and replication of the fungal cells [57].

#### 3.4.3. Protective Effect of Gold Nanoparticle GNP023 against ABTS Radical in Antioxidant Assay and Hydroxyl Radical in DNA Nick Assay

**Figure 10:**
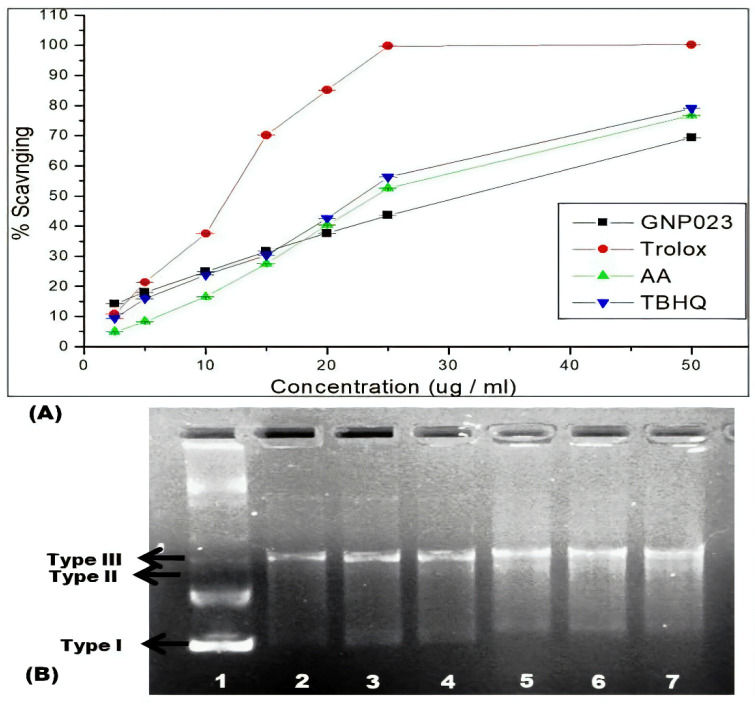
(**A**) ABTS antioxidant activity of the gold nanoparticle GNP023, in comparison to controls and standards Trolox, Ascorbic acid and TBHQ. (**B**) Development of a pBSK DNA nicking assay with gold nanoparticle GNP023. Lane 1—Native pBSK DNA; Lane 2—pBSK DNA with Fenton’s reagent; Lane 3—pBSK DNA with 25 μg/mL Curcumin and Fenton reagent; and Lanes 4 to 7—pBSK DNA with Fenton’s reagent and GNP023 (25–200 μg/mL, respectively) (Type I—closed circular DNA forms; Type II/III—open circular DNA forms).

The antioxidant activity of the gold nanoparticle GNP023 was determined, using the half-maximal inhibitory concentration IC_50_. Radical-scavenging activity of the gold nano-particle GNP023 against ABTS+ radical is illustrated in Figure 10A. The IC_50_ value revealed significant antioxidant activity, with an IC_50_ of 38.61 µg/mL against ABTS+ radical, which is similar to the results of Adebayo et al. [58].

The generation of highly reactive oxidizing hydroxyl radical changes the configuration of plasmid DNA from circular to nicked linear forms and/or open circular, thus affecting the electrophoretic mobility of the DNA. To understand the potential ability of the GNP023 in inhibiting the DNA damage against oxidative stress, a DNA nicking assay was employed [59]. DNA protection can be assessed in terms of the supercoiled form of plasmid DNA, after treatment with different concentrations of GNP023. Figure 10B displays the concentration-dependent intensification of native supercoiled DNA. The supercoiled plasmid DNA (Lane 1, Type 1) was degraded into single and/or open circular DNA forms (Lane 2, Type II and III), due to the generation of highly reactive hydroxyl radicals. Curcumin (25 μg/mL) exhibited protection of supercoiled form of the DNA (Lane 3). A similar trend was observed for the GNP023 treatment, with varying concentrations (Lane 4–7, 200–25 μg/mL, respectively). A dose-dependent plasmid DNA protection was observed in our study. Whereas, when AREF023 methanol extract was subjected to this assay (provided as a supplementary material—Figure S3); the supercoiled DNA (Lane 4–6) was not protected, thus verifying the inability of the extract to inhibit DNA damage against the oxidative stress.

#### 3.4.4. Cell Cytotoxicity/Viability Assay of the Gold Nanoparticle GNP023

**Figure 11:**
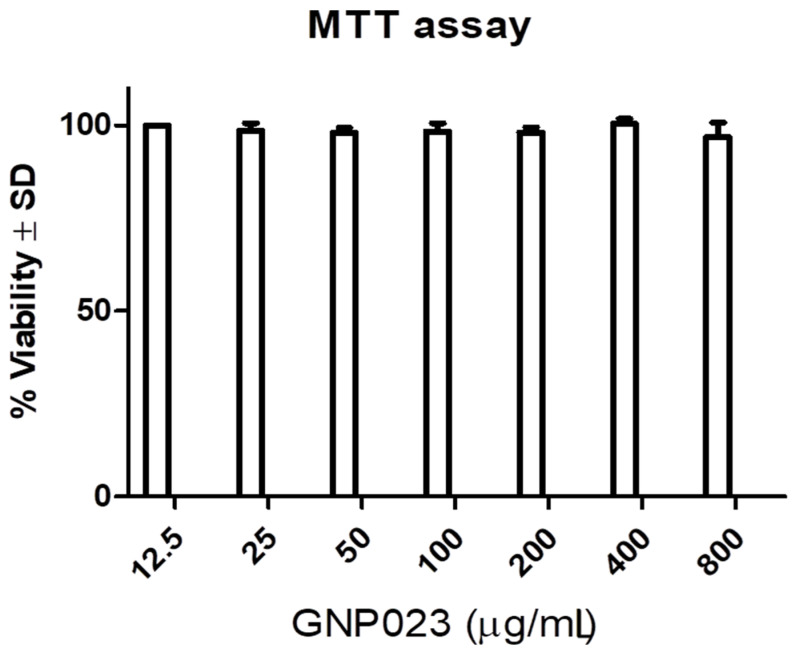
Effect of gold nanoparticle GNP023 on HEK 293 T normal cell line.

The toxicity of gold nanoparticle GNP023 was analysed by checking the viability on HEK293 T cells, by employing the in vitro MTT assay, as described in the Materials and Methods section. The results indicate that cells post-GNP023 treatment, with a range of concentrations (12–800 µg/mL), displayed insignificant cell death, indicating low or no toxicity for these nanoparticles. The results obtained were plotted as a bar graph (Figure 11), illustrating the percent difference between GNP023 for treated versus untreated cells, for evaluation of cellular viability. Our findings are in good agreement with a recently published report [60]. We also checked the AREF023 methanol extracts’s cytotoxic effect (i.e., cell death), as compared to control cells (provided as a supplementary material—Figure S4) at varying concentrations (12.5 µg/mL to 1600 µg/mL), suggesting it is unsafe for application in host cells.

## 4. Conclusions

Past studies have established the usefulness of endophytic fungi in the biosynthesis of bioactive compounds. However, gold nanoparticle production by endophytic fungi has not been, extensively, explored. Here, we report on the bio-fabrication of gold nanoparticles, using the endophytic fungus *A. terreus,* obtained from *D. metel*. To the best of our knowledge, this is the first report on the antioxidant and antimicrobial potential of GNPs synthesised using this endophytic fungus. These GNPs were characterised using UV-visible, FTIR, TEM, EDX, XRD and DLS analysis. The synthesised GNPs were 100 nm or less in size and exhibited either antioxidant or antifungal/antimicrobial activity against food-borne pathogens. These nanoparticles were, also, nontoxic to human embryonic kidney cells, at measured concentrations from 400 µg/mL to 800 µg/mL. LC–MS analysis showed the presence of fungal enzymatic amino peptides as well as phenolic and carbamate compounds in the methanolic extract of *A. terreus,* which are important components for the reduction and stabilization of biosynthesised gold nanoparticles. FTIR analysis confirmed the presence of carboxylic acid, phenolic aicd and amide linkages, which are important functional groups for the stabilization and capping of gold nanoparticles. These results indicated which functional groups were involved in the reduction and capping of biosynthesised nanoparticles, which indicated that specific compounds can be identified that play an important role in the endogenous formation of GNPs from fungi.

## Figures and Tables

**Table 1:** Determination of bioactive compounds in extract of *A. terreus* AREF023 by LC-ESI-MS (+ion mode).

Analyte No.	Tentative Allotment of Compounds Based on METLIN	Chemical Formula	Parent Ion (m/z) (Positive Ion Mode [M + H]	Peak Intensity	Mass Recorded (METLIN)	METLIN ID
1	Levothyroxine sodium anhydrous	C_15_H_11_I_4_NO_4_	799.6769	8.05 × 10^5^	798.6686	66850
2	Sitoindoside I	C_51_H_90_O_7_	815.6778	1.84 × 10^6^	814.6687	87222
3	3,3′-Difluoro-4-[(6-methyloctyl)oxy]-4′-undecyl-1,1′-biphenyl	C_32_H_48_F_2_O	487.3727	3.81 × 10^6^	486.3673	894417
4	Flavidulol C	C_34_H_42_O_4_	515.3141	6.69 × 10^5^	514.3083	93747
5	Capsicoside C3	C_44_H_70_O_17_	871.4661	1.49 × 10^6^	870.4613	95394
6	tert-Butyl {5-[6-(benzyloxy)naphthalen-2-yl]-2,2-dimethyl-1,3-dioxan-5-yl}carbamate	C_28_H_33_NO_5_	464.2419	1.60 × 10^6^	463.2359	862873
7	N-(2-Hydroxyethyl)-3,5-bis(octadecyloxy)benzamide	C_45_H_83_NO_4_	702.6394	1.36 × 10^5^	701.6322	909645

**Table 2:** Antimicrobial activity of gold nanoparticle GNP023.

	Diameter of Zone of Inhibition (mm) * against Pathogenic Bacteria			
Concentrations (µg/mL)	*S. aureus*	*S. typhimurium*	*Methicillin resistant S. aureus*	*V. cholerae*
DMSO (control)	0	0	0	0
GNP023—100	0	0	0	5.2 ± 0.18
GNP023—200	4.1 ± 0.07	0	0	6.1 ± 0.12
GNP023—400	8.58 ± 0.28	0	6.1 ± 0.12	9.31 ± 0.14
Vancomycin (25 µg/ disk)	10.06 ± 0.16	-	9.53 ± 0.23	-
Chloramphenicol (25 µg/disk)	-	10.51 ± 0.23	-	10.53 ± 0.21

*: mean diameter on zone of inhibition ± Standard Deviation (*n* = 3). -: indicates not applied.

**Table 3:** Antifungal activity of GNP023 on in vitro mycelia growth of plant pathogenic fungi.

Pathogenic Fungal Strain	Percentage Inhibition (%) * Mycelial Growth after GNP023 (µg/mL) Treatment				
	Control	25 µg/mL	50 µg/mL	100 µg/mL	200 µg/mL
*Rhizoctonia solani*	0	10.75 ± 0.22	31.96 ± 0.42	36.24 ± 0.55	52.5 ± 0.47
*Fusarium oxysporum*	0	18.28 ± 0.46	34.51 ± 0.38	56.30 ± 0.45	65.46 ± 0.37

*: mean diameter on zone of inhibition ± Standard Deviation (*n* = 3).

## Data Availability

Most of the data is provided in the manuscript and supplementary material. Data presented in this study are available on request from the corresponding author.

## Supplementary Materials

The following supporting information can be downloaded at: https://www.mdpi.com/article/10.3390/ma15113877/s1, Figure S1. Zones of inhibition produced by AREF023 Methanol extract (ME), against bacterial pathogens (A)—S. aureus, (B)—S. typhimurium, (C)—Methicillin-resistant S. aureus (MRSA and; (D)—V. cholerae. [V—Vancomycin; Ch—Chloramphenicol; 400—AREF023 ME 400 µg/ml; 600—AREF023 ME 600 µg/ml; 800AREF023 ME 800 µg/ml; C— Control]. Figure S2. Antifungal activity of AREF023 methanol extract against plant pathogenic fungi (a) Rhizoctonia solani and (b) Fusarium oxysporum. PDA plates were amended with different concen-trations of AREF023 ME (i) control, (ii) 200 μg/mL, and (iii) 400 μg/mL of methanol extract of AREF023 strain. Figure S3. Development of a pBSK DNA nicking assay with A. terreus AREF023 methanol extract. Lane 1—Native pBSK DNA, Lane 2—pBSK DNA with Fenton’s reagent, Lane 3—pBSK DNA with 25 μg/ml Curcumin and Fenton reagent, and Lanes 4 to 6—pBSK DNA with Fen-ton’s reagent and AREF023 ME (100–300 μg/ml, respectively). Figure S4. Effect of AREF023 methanol extract on HEK 293T normal cell line. Table S1. Antimicrobial activities of AREF023 methanol extract (ME). Table S2: Antifungal activity of AREF023 methanol extract on in vitro mycelia growth of plant pathogenic fungi.

## Abbreviations

ABTS	2,2′-azino-bis (3-ethylbenzothiazoline-6-sulfonic acid
EDX	Energy-dispersive X-ray
FTIR	Fourier-Transform Infrared GNP–gold nanoparticle
MTT	3-[4,5-dimethylthiazol-2-yl]-2,5 diphenyl tetrazolium bromide NB–nutrient broth
SPR	Surface Plasmon Resonance
TEM	Transmission electron microscopy
XRD	X-ray diffraction

## References

[1] Elegbede J.A., Lateef A., Azeez M.A., Asafa T.B., Yekeen T.A., Oladipo I.C., Aina D.A., Beukes L.S., Gueguim-Kana E.B. (2020). Biofabrication of gold nanoparticles using xylanases through valorization of corncob by Aspergillus niger and Trichoderma longibrachiatum: Antimicrobial, antioxidant, anticoagulant and thrombolytic activities. Waste Biomass Valorization.

[2] Ojo S.A., Lateef A., Azeez M.A., Oladejo S.M., Akinwale A.S., Asafa T.B., Yekeen T.A., Akinboro A., Oladipo I.C., Gueguim-Kana E.B. (2016). Biomedical and catalytic applications of gold and silver-gold alloy nanoparticles biosynthesized using cell-free extract of Bacillus safensis LAU 13: Antifungal, dye degradation, anti-coagulant and thrombolytic activities. IEEE Trans. Nanobiosci..

[3] Sharma R., Singh D., Singh R. (2009). Biological control of postharvest diseases of fruits and vegetables by microbial antagonists: A review. Biol. Control..

[4] Eng S.-K., Pusparajah P., Mutalib N.-S.A., Ser H.-L., Chan K.-G., Lee L.-H. (2015). Salmonella: A review on pathogenesis, epidemiology and antibiotic resistance. Front. Life Sci..

[5] Välimaa A.-L., Tilsala-Timisjärvi A., Virtanen E. (2015). Rapid detection and identification methods for Listeria monocytogenes in the food chain—A review. Food Control..

[6] Heiman K.E., Mody R.K., Johnson S.D., Griffin P.M., Hannah Gould L. (2015). Escherichia coli O157 outbreaks in the United States, 2003–2012. Emerg. Infect. Dis..

[7] Kothary M.H., Babu U.S. (2001). Infective dose of foodborne pathogens in volunteers: A review. J. Food Saf..

[8] Chukwu E.E., Nwaokorie F.O., Coker A.O., Avila-Campos M.J., Solis R.L., Llanco L.A., Ogunsola F.T. (2016). Detection of toxigenic Clostridium perfringens and Clostridium botulinum from food sold in Lagos, Ni geria. Anaerobe.

[9] Deacon J.W., Berry L.A. (1993). Biocontrol of soil-borne plant pathogens: Concepts and their application. Pestic. Sci..

[10] Mishra R.C., Goel M., Colin B., Deshmukh S.K. (2020). Endophytic fungi-an untapped source of potential antioxidants. Curr. Bioact. Compd..

[11] Mishra R.C., Kalra R., Dwivedi N., Goel M. (2021). Exploring endophytes using “Omics”: An approach for sustainable production of bioactive metabolites. Mycoremediation and Environmental Sustainability.

[12] Rai A., Prabhune A., Perry C.C. (2010). Antibiotic mediated synthesis of gold nanoparticles with potent antimicrobial activity and their application in antimicrobial coatings. J. Mater. Chem..

[13] Zawrah M., El-Moez S., Center D. (2011). Antimicrobial activities of gold nanoparticles against major foodborne pathogens. Life Sci. J..

[14] Patra P., Shouvik M., Nitai D., Arunava G. (2012). Biochemical-, biophysical-, and microarray-based antifungal evaluation of the buffer-mediated synthesized nano zinc oxide: An in vivo and in vitro toxicity study. Langmuir.

[15] Win T.T., Khan S., Fu P. (2020). Fungus-(*Alternaria* sp.) mediated silver nanoparticles synthesis, characterization, and screening of antifungal activity against some phytopathogens. J. Nanotechnol..

[16] Samrot A.V., Bhavya K.S., Sahithya C.S., Sowmya N. (2018). Evaluation of toxicity of chemically synthesised gold nanoparticles against Eudrilus eugeniae. J. Clust. Sci..

[17] Chen Y.-S., Hung Y.-C., Liau I., Huang G.S. (2009). Assessment of the in vivo toxicity of gold nanoparticles. Nanoscale Res. Lett..

[18] Clement J.L., Jarrett P.S. (1994). Antibacterial silver. Met. -Based Drugs.

[19] Park H.-J., Kim S.-H., Kim H.-J., Seong C. (2006). A new composition of nanosized silica-silver for control of various plant diseases. Plant Pathol. J..

[20] Mukherjee P., Ahmad A., Mandal D., Senapati S., Sainkar S.R., Khan M.I., Ramani R., Parischa R., Ajayakumar P.V., Alam M. (2001). Bioreduction of AuCl4− ions by the fungus, Verticillium sp. and surface trapping of the gold nanoparticles formed. Angew. Chem. Int. Ed..

[21] Asharani P.V., Yi L., Gong Z., Valiyaveettil S. (2011). Comparison of the toxicity of silver, gold and platinum nanoparticles in developing zebrafish embryos. Nanotoxicology.

[22] Qadri M., Johri S., Shah B., Khajuria A., Sidiq T., Lattoo S., Abdin M., Riyaz-Ul-Hassan S. (2013). Identification and bioactive potential of endophytic fungi isolated from selected plants of the Western Himalayas. SpringerPlus.

[23] Zhao J., Fu Y., Luo M., Zu Y., Wang W., Zhao C., Gu C. (2012). Endophytic fungi from pigeon pea [Cajanus cajan (L.) Millsp.] produce antioxidant cajaninstilbene acid. J. Agric. Food Chem..

[24] Mishra R.C., Kalra R., Dilawari R., Deshmukh S.K., Barrow C.J., Goel M. (2021). Characterization of an endophytic strain Talaromyces assiutensis, CPEF04 with evaluation of production medium for extracellular red pigments having antimicrobial and anticancer properties. Front. Microbiol..

[25] CLSI (2015). Performance Standards for Antimicrobial Susceptibility Testing.

[26] Aguilar-Méndez M.A., Martín-Martínez E.S., Ortega-Arroyo L., Cobián-Portillo G., Sánchez-Espíndola E. (2011). Synthesis and characterization of silver nanoparticles: Effect on phytopathogen Colletotrichum gloesporioides. J. Nanoparticle Res..

[27] Re R., Pellegrini N., Proteggente A., Pannala A., Yang M., Rice-Evans C. (1999). Antioxidant activity applying an improved ABTS radical cation decolorization assay. Free. Radic. Biol. Med..

[28] Jiang X., Lu C., Tang M., Yang Z., Jia W., Ma Y., Jia P., Pei D., Wang H. (2018). Nanotoxicity of silver nanoparticles on HEK293T cells: A combined study using biomechanical and biological techniques. ACS Omega.

[29] Sunkar S., Nachiyar V. (2013). Endophytes as potential nanofactories. Int. J. Chem. Environ. Biol. Sci..

[30] Abdel-Raouf N., Al-Enazi N.M., Ibraheem I.B. (2017). Green biosynthesis of gold nanoparticles using Galaxaura elongata and characterization of their antibacterial activity. Arab. J. Chem..

[31] Ahmad A., Wei Y., Syed F., Imran M., Khan Z.U.H., Tahir K., Khan A.U., Raza M., Khana Q., Yuan Q. (2015). Size dependent catalytic activities of green synthesized gold nanoparticles and electro-catalytic oxidation of catechol on gold nanoparticles modified electrode. RSC Adv..

[32] Mulvaney P. (1996). Surface plasmon spectroscopy of nanosized metal particles. Langmuir.

[33] Binupriya A.R., Sathishkumar M., Vijayaraghavan K., Yun S.-I. (2010). Bioreduction of trivalent aurum to nano-crystalline gold particles by active and inactive cells and cell-free extract of Aspergillus oryzae var. viridis. J. Hazard. Mater..

[34] Gade A.K., Bonde P.P., Ingle A., Marcato P.D. (2008). Exploitation of Aspergillus niger for synthesis of silver nanoparticles. J. Biobased Mater. Bioenergy.

[35] Bhainsa K.C., D’souza S. (2006). Extracellular biosynthesis of silver nanoparticles using the fungus Aspergillus fumigatus. Colloids Surf. B Biointerfaces.

[36] Parial D., Patra H.K., Dasgupta A.K., Pal R. (2012). Screening of different algae for green synthesis of gold nanoparticles. Eur. J. Phycol..

[37] Singh A.K., Talat M., Singh D.P., Srivastava O.N. (2010). Biosynthesis of gold and silver nanoparticles by natural precursor clove and their functionalization with amine group. J. Nanopart. Res..

[38] Ke Y., Aboody M.S.A., Alturaiki W., Alsagaby S.A., Alfaiz F.A., Veeraraghavan V.P., Mickymaray S. (2019). Photosynthesized gold nanoparticles from Catharanthus roseus induces caspase-mediated apoptosis in cervical cancer cells (HeLa). Artif. Cells Nanomed. Biotechnol..

[39] Vigneshwaran N., Ashtaputre N.M., Varadarajan P.V., Nachane R.P., Paralikar K.M., Balasubramanya R.H. (2007). Biological synthesis of silver nanoparticles using the fungus Aspergillus flavus. Mater. Lett..

[40] Sujitha M.V., Kannan S. (2013). Green synthesis of gold nanoparticles using Citrus fruits (*Citrus limon, Citrus reticulata* and *Citrus sinensis*) aqueous extract and its characterization. Spectrochim. Acta Part A Mol. Biomol. Spectrosc..

[41] Song J.Y., Jang H.-K., Kim B.S. (2009). Biological synthesis of gold nanoparticles using Magnolia kobus and Diopyros kaki leaf extracts. Process Biochem..

[42] Verma V.C., Singh S.K., Solanki R., Prakash S. (2011). Biofabrication of anisotropic gold nanotriangles using extract of endophytic Aspergillus clavatus as a dual functional reductant and stabilizer. Nanoscale Res. Lett..

[43] Varghese S., Akshaya C., Jisha M. (2021). Unravelling the bioprospects of mycoendophytes residing in withania somnifera for productive pharmaceutical applications. Biocatal. Agric. Biotechnol..

[44] Aswani P., Tijith K., George T., Shanavas J. (2017). Characterization of bioactive metabolites of endophytic Fusarium solani isolated from Withania somnifera. J. Biol. Act. Products Nat..

[45] Fujimoto H., Nakayama Y., Yamazaki M. (1993). Identification of immunosuppressive components of a mushroom, Lactarius flavidulus. Chem. Pharm. Bull..

[46] Chávez M.I., Soto M., Taborga L., Díaz K., Olea A.F., Bay C., Peña-Cortés H., Espinoza L. (2015). Synthesis and in vitro antifungal activity against Botrytis cinerea of geranylated phenols and their phenyl acetate derivatives. Int. J. Mol. Sci..

[47] Iorizzi M., Lanzotti V., Ranalli G., De Marino S., Zollo F. (2002). Antimicrobial furostanol saponins from the seeds of Capsicum annuum L. var. acuminatum. J. Agric. Food Chem..

[48] de Rodríguez D.J., Angulo-Sánchez J.L., Hernández-Castillo F.D. (2006). An overview of the antimicrobial properties of Mexican medicinal plants. Adv. Phytomed..

[49] Ibrahim S.R.M., Mohamed G.A., Haidari R.A.A., Zayed M.F., El-Kholy A.A., Elkhayat E.S., Ross S.A. (2018). Fusarithioamide B, a new benzamide derivative from the endophytic fungus Fusarium chlamydosporium with potent cytotoxic and antimicrobial activities. Bioorganic Med. Chem..

[50] Bauer A.W., Kirby W.M., Sherris J.C., Turck M. (1966). Antibiotic susceptibility testing by a standardized single disc method. Am. J. Clin. Pathol..

[51] Chamakura K., Perez-Ballestero R., Luo Z., Bashir S., Liu J. (2011). Comparison of bactericidal activities of silver nanoparticles with common chemical disinfectants. Colloids Surf. B Biointerfaces.

[52] Morones J.R., Elechiguerra J.L., Camacho A., Holt K., Kouri J.B., Ramírez J.T., Yacaman M.J. (2005). The bactericidal effect of silver nanoparticles. Nanotechnology.

[53] Carlson C., Hussain S.M., Schrand A.M., Braydich-Stolle L.K., Hess K.L., Jones R.L., Schlager J.J. (2008). Unique cellular interaction of silver nanoparticles: Size-dependent generation of reactive oxygen species. J. Phys. Chem. B.

[54] Park H.-J., Kim J.Y., Kim J., Lee J.-H., Hahn J.-S., Gu M.B., Yoon J. (2009). Silver-ion-mediated reactive oxygen species generation affecting bactericidal activity. Water Res..

[55] Zada S., Ahmad A., Khan S., Iqbal A., Ahmad S., Ali H., Fu P. (2018). Biofabrication of gold nanoparticles by Lyptolyngbya JSC-1 extract as super reducing and stabilizing agents: Synthesis, characterization and antibacterial activity. Microb. Pathog..

[56] Wani I.A., Ahmad T. (2013). Size and shape dependant antifungal activity of gold nanoparticles: A case study of Candida. Colloids Surf. B Biointerfaces.

[57] Beyenbach K.W., Wieczorek H. (2006). The V-type H+ ATPase: Molecular structure and function, physiological roles and regulation. J. Exp. Biol..

[58] Tan Y.N., Lee K.H., Su X. (2011). Study of single-stranded DNA binding protein-nucleic acids interactions using unmodified gold nanoparticles and its application for detection of single nucleotide polymorphisms. Anal. Chem..

[59] Adebayo A.E., Oke A.M., Lateef A., Oyatokun A.A., Abisoye O.D., Adiji I.P., Fagbenro D.O., Amusan T.V., Badmus J.A., Asafa T.B. (2019). Biosynthesis of silver, gold and silver–gold alloy nanoparticles using Persea americana fruit peel aqueous extract for their biomedical properties. Nanotechnol. Environ. Eng..

[60] Leba L.-J., Brunschwig C., Saout M., Martial K., Vulcain E., Bereau D., Robinson J.-C. (2014). Optimization of a DNA nicking assay to evaluate Oenocarpus bataua and Camellia sinensis antioxidant capacity. Int. J. Mol. Sci..

