# Supplementary material for: Bio-Synthesis of Aspergillus terreus Mediated Gold Nanoparticle: Antimicrobial, Antioxidant, Antifungal and In Vitro Cytotoxicity Studies
Source: Materials (Basel). 2022 May 29;15(11):3877. doi: 10.3390/ma15113877 (PMC9181662; doi:10.3390/ma15113877)
Supplement: Supplementary file 1 [file materials-15-03877-s001.zip › materials-1709303-supplementary.pdf]

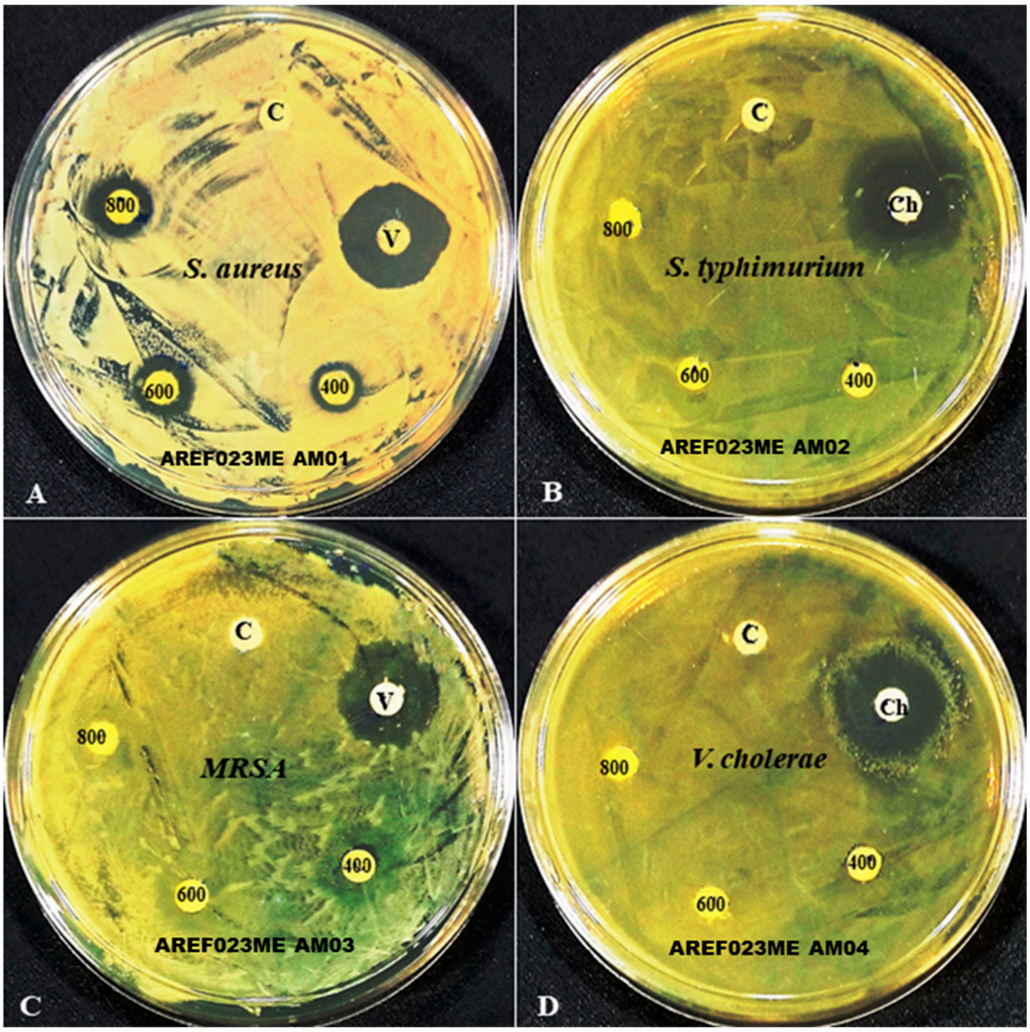

Figure S1. Zones of inhibition produced by AREF023 Methanol extract (ME), against bacterial pathogens (**A**)—*S. aureus*, (**B**)—*S. typhimurium*, (**C**)—Methicillin-resistant *S. aureus* (MRSA and; (**D**)—*V. cholerae*. [V—Vancomycin; Ch—Chloramphenicol; 400—AREF023 ME 400 µg/ml; 600—AREF023 ME 600 µg/ml; 800AREF023 ME 800 µg/ml; C— Control].

Table S1. Antimicrobial activities of AREF023 methanol extract (ME).

Concentrations (µg/ml)	Diameter of zone of inhibition (mm)* against pathogenic bacteria			
	S. aureus	*S. typhimurium*	*Methicillin resistant S. aureus*	*V. cholerae*
DMSO (control)	0	0	0	0
AREF023 ME 400	4.03 ± 0.15	0	0	0
AREF023 ME 600	5.27 ± 0.24	0	0	0
AREF023 ME 800	6.42 ± 0.39	0	0	0
Vancomycin (25 µg/ disk)	8.83 ± 0.28	-	8.63 ± 0.23	-
Chloramphenicol (25 µg/ disk)	-	7.28 ± 0.25	-	8.31 ± 0.21

*: mean diameter on zone of inhibition ± Standard Deviation (n = 3). -: indicates not applied.

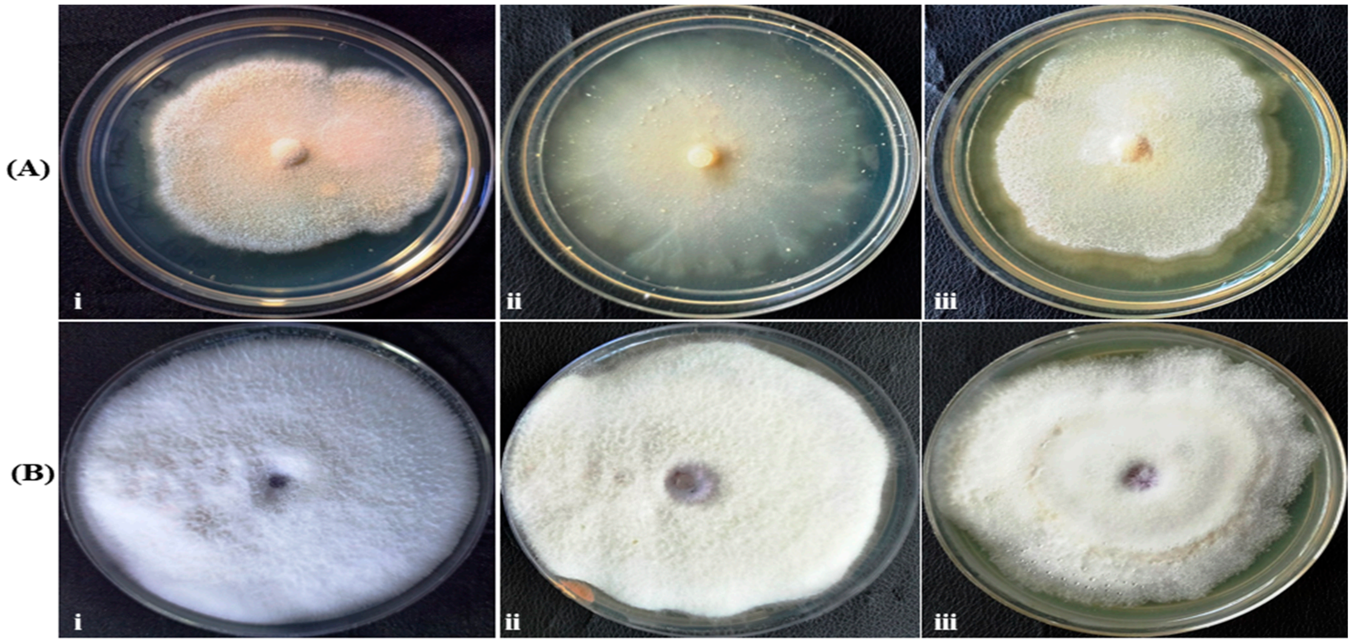

Figure S2. Antifungal activity of AREF023 methanol extract against plant pathogenic fungi (**A**) *Rhizoctonia solani* and (**B**) *Fusarium oxysporum*. PDA plates were amended with different concentrations of AREF023 ME (i) control, (ii) 200µg/mL, and (iii) 400µg/mL of methanol extract of AREF023 strain.

Table S2. Antifungal activity of AREF023 methanol extract on in vitro mycelia growth of plant pathogenic fungi.

Pathogenic fungal strain	Percentage Inhibition (%)* mycelial growth after AREF023 ME (µg/ml) treatment		
	Control	200 µg/ml	400 µg/ml
Rhizoctonia solani	0	3.76 ± 0.3	9.49 ± 0.34
Fusarium oxysporum	0	2.51 ± 0.21	4.64 ± 0.28

*: mean % inhibition of fungal mycelium ± Standard Deviation (n = 3).

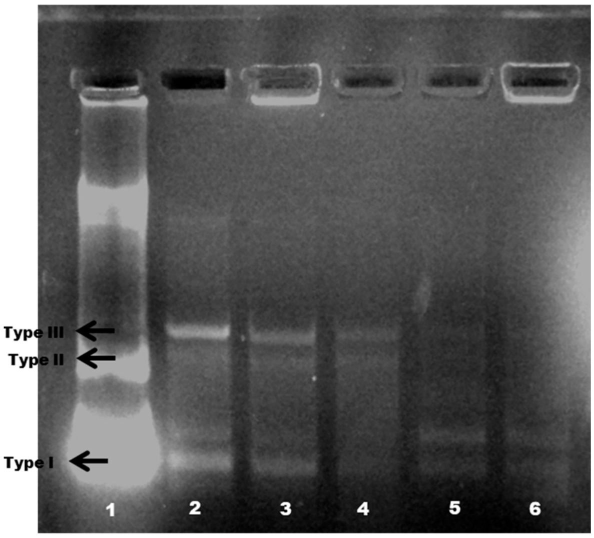

Figure S3. Development of a pBSKDNA nicking assay with *A. terreus* AREF023 methanol extract. Lane 1—Native pBSK DNA, Lane 2—pBSK DNA with Fenton's reagent, Lane 3—pBSK DNA with 25 µg/ml Curcumin and Fenton reagent, and Lanes 4 to 6—pBSK DNA with Fenton's reagent and AREF023 ME (100–300 µg/ml, respectively).

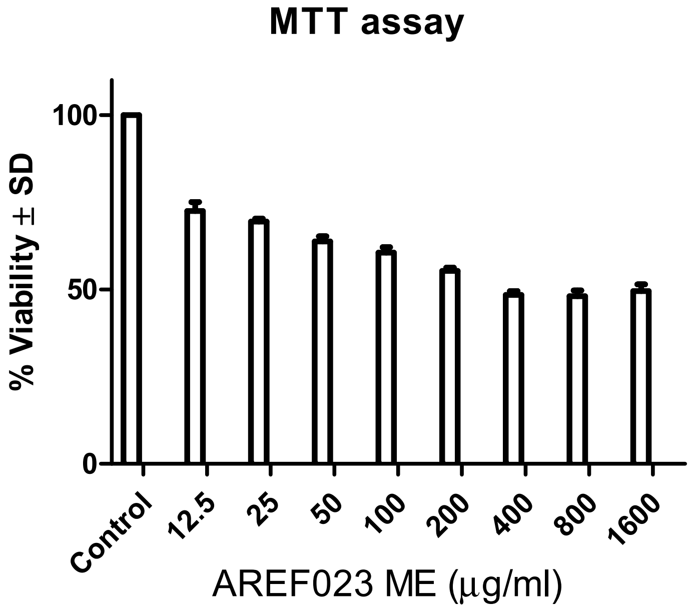

Figure S4. Effect of AREF023 methanol extract on HEK 293T normal cell line.
